# ‘New Medicine Service’: supporting adherence in people starting a new medication for a long-term condition: 26-week follow-up of a pragmatic randomised controlled trial

**DOI:** 10.1136/bmjqs-2018-009177

**Published:** 2019-11-15

**Authors:** Rachel Ann Elliott, Matthew J Boyd, Lukasz Tanajewski, Nick Barber, Georgios Gkountouras, Anthony J Avery, Rajnikant Mehta, James E Davies, Nde-Eshimuni Salema, Christopher Craig, Asam Latif, Justin Waring, Antony Chuter

**Affiliations:** 1 Manchester Centre for Health Economics, School of Health Sciences, University of Manchester, Manchester, UK; 2 Division of Pharmacy Practice and Policy, University of Nottingham School of Pharmacy, Nottingham, UK; 3 Department of Economics, Kozminski University, Warsaw, Poland; 4 School of Pharmacy, UCL School of Pharmacy, London, UK; 5 Primary Care, University of Nottingham, Nottingham, UK; 6 School of Medicine and Dentistry, University of Birmingham, Birmingham, UK; 7 School of Pharmacy, Health Bridge, London, UK; 8 School of Medicine, University of Nottingham, Nottingham, UK; 9 Institute of Mental Health, CLAHRC East Midlands, Nottingham, UK; 10 School of Health Sciences, University of Nottingham, Nottingham, UK; 11 Nottingham University Business School, University of Nottingham, Nottingham, UK; 12 School of Pharmacy, 68 Brighton Cottages, Haywards Heath, UK

**Keywords:** compliance, cost-effectiveness, decision analysis, randomised controlled trial, pharmacists

## Abstract

**Objective:**

To examine the effectiveness and cost-effectiveness of the community pharmacy New Medicine Service (NMS) at 26 weeks.

**Methods:**

Pragmatic patient-level parallel randomised controlled trial in 46 English community pharmacies. 504 participants aged ≥14, identified in the pharmacy when presenting a prescription for a new medicine for predefined long-term conditions, randomised to receive NMS (n=251) or normal practice (n=253) (NMS intervention: 2 consultations 1 and 2 weeks after prescription presentation). Adherence assessed through patient self-report at 26-week follow-up. Intention-to-treat analysis employed. National Health Service (NHS) costs calculated. Disease-specific Markov models estimating impact of non-adherence combined with clinical trial data to calculate costs per extra quality-adjusted life-year (QALY; NHS England perspective).

**Results:**

Unadjusted analysis: of 327 patients still taking the initial medicine, 97/170 (57.1%) and 103/157 (65.6%) (p=0.113) patients were adherent in normal practice and NMS arms, respectively. Adjusted intention-to-treat analysis: adherence OR 1.50 (95% CI 0.93 to 2.44, p=0.095), in favour of NMS. There was a non-significant reduction in 26-week NHS costs for NMS: −£104 (95% CI −£37 to £257, p=0.168) per patient. NMS generated a mean of 0.04 (95% CI −0.01 to 0.13) more QALYs per patient, with mean reduction in lifetime cost of −£113.9 (−1159.4, 683.7). The incremental cost-effectiveness ratio was −£2758/QALY (2.5% and 97.5%: −38 739.5, 34 024.2. NMS has an 89% probability of cost-effectiveness at a willingness to pay of £20 000 per QALY.

**Conclusions:**

At 26-week follow-up, NMS was unable to demonstrate a statistically significant increase in adherence or reduction in NHS costs, which may be attributable to patient attrition from the study. Long-term economic evaluation suggested NMS may deliver better patient outcomes and reduced overall healthcare costs than normal practice, but uncertainty around this finding is high.

**Trial registration number:**

NCT01635361, ISRCTN23560818, ISRCTN23560818, UKCRN12494.

## Introduction

The New Medicine Service (NMS) introduced in England in 2011^1^ supports people starting a newly initiated medication for a long-term condition in four specified patient groups associated with high rates of avoidable hospital admissions (asthma/chronic obstructive pulmonary disease, hypertension, type 2 diabetes, or prescription of an anticoagulant/antiplatelet agent). NMS is based on our previous work in developing and testing an intervention targeting poor medicine adherence in people receiving a new medicine for a long-term condition.^2–4^ NMS is delivered by the community pharmacist supplying the medicine, either face-to-face or over the telephone. When people start a new medicine, they often experience problems which can lead to a significant proportion becoming non-adherent.^5^ NMS provides a starting point for the pharmacists to resolve individuals’ specific problems with information and advice. Accredited pharmacies provide NMS, are remunerated for each episode of care and have guidance delivering the intervention.^6^

We have already demonstrated that the NMS increases the proportion of patients reporting adherence to their new medicine 10 weeks after the intervention, by 10.2%.^7^ In an economic analysis modelling impact of adherence changes on health status and cost over lifetime, NMS was more effective and less costly than normal practice.^8^ NMS demonstrated a 96.7% probability of cost-effectiveness compared with normal practice at a willingness to pay of £20 000 per quality-adjusted life-year (QALY).

As a result of this work based on effectiveness at 10-week follow-up, NMS was approved for routine commissioning in the National Health Service (NHS) in England.^9 10^ New services with similar configurations, or explicitly based on NMS, have since been trialled or set up in other settings: Scotland (New Medicine Intervention Support Tools)^11^; Australia (New Medicines Support Service)^12^; Norway (Medicines Startup-Medisinstart)^13^; Ireland (NMS)^14^; and Belgium (Begeleidingsgesprek Nieuwe Medicatie^15^ and Entretien d’accompagnement de Nouvelle Médication).^16^

In the original trial we also measured self-reported adherence at 26-week follow-up as a secondary outcome to assess persistence of effect over time. This was in response to the lack of evidence around longer term effectiveness of interventions intended to improve adherence. If there was a reasonable degree of persistence of the effect at 26 weeks then health gains would be increased. If reasonably effective at 6 months then the approach could be incorporated into existing six monthly reviews of medication in the NHS, providing a continuous monitoring and feedback loop to improve patients’ lives. In this paper, we ask the extent to which the adherence improvement and cost-effectiveness observed at 10 weeks were maintained at 26 weeks.

## Methods

### Study design

The study was a patient-level multicentre, pragmatic randomised controlled trial (RCT) with parallel group design,^17 18^ reported according to Consolidated Standards of Reporting Trials (CONSORT) criteria.^19^

### Study setting

Community pharmacies in East Midlands and South Yorkshire and Greater London accredited to provide NMS. Pharmacy selection took into account pharmacy ownership, proximity to general practice (GP), setting and economic deprivation. (See online supplementary appendix 1 for details.)

10.1136/bmjqs-2018-009177.supp1 Supplementary data

### Study participants

Patients could participate if they were eligible for NMS, community dwelling, aged 14 or over and able to consent to the NMS and the study (parental consent for 14–15 year-olds).

### Pharmacy and patient recruitment

Pharmacies from all groups covering the range of characteristics in the setting criteria above were invited to participate, those initiating at least two NMS consultations/week were recruited. Of 61 recruiting pharmacies, 46 (75.4%) provided patients. No further training on delivering the intervention or normal practice was provided (see online supplementary appendix 1).

Patients were recruited within community pharmacies by pharmacists trained about the study. The process and outcome of being randomised was explained to patients. They were given as long as they needed to read the study information and ask questions.

### Randomisation and blinding

Patients were randomised into one of the two study arms, using sealed envelopes, stratified by drug/disease group using Statistical Analysis Software (SAS V.9.3).^20^ Block randomisation was used within each pharmacy to avoid allocation imbalances. Researchers collecting data were blinded to study arm.

### NMS intervention

NMS begins with the patient’s presentation at a community pharmacy with a prescription for a new medicine. The NMS intervention comprises two parts, which can be face-to-face or over the telephone, named ‘intervention’ and ‘follow-up’. The pharmacist invites the patient to a one-to-one consultation 7–14 days later (the ‘intervention’) with a ‘follow-up’ 14–21 days after that. These are the points in the service at which the pharmacist would ask about adherence. The NMS intervention should be completed in a maximum of 5 weeks (online supplementary appendix 2).

### Normal practice

Normal practice was the pharmacist’s usual advice when presented with a prescription for a new medicine for a long-term condition. No follow-up was offered to these patients.

### Primary (10-week adherence) and secondary outcomes (26-week adherence)

The study was powered to detect a difference in self-reported non-adherence at 10 weeks’ follow-up, as agreed with the funder. The study was not explicitly powered to detect a difference at 26 weeks but we collected the same outcome at 26 weeks, as a secondary outcome, to assess persistence of effect over time.

Adherence was assessed by telephone at 10 and 26 weeks using the same adherence measure used by pharmacists in the NMS, the ‘NMS question’, a question similar to the question in our original work^3^: ‘People often miss taking doses of their medicines, for a wide range of reasons. Have you missed any doses of your new medicine, or changed when you take it? (Prompt: when did you last miss a dose?).’^21^

The patient was defined as non-adherent if any doses were missed without agreement with a medical professional in the previous 7 days. A sample size of 200 patients/arm was required to detect decreased non-adherence from 20% to 10% with 80% power, 5% significance level (two tailed). Up to 100 patients were expected to be lost to follow-up, withdraw, or change/stop medication by 10 weeks, giving a required sample of 250 patients/arm.

### Other medicine-taking outcomes

The Morisky Eight-Item Medication Adherence Scale (MMAS-8), validated in hypertension, was used with permission, to support our primary outcome measure, and collected via self-completion postal questionnaire at 10 and 26 weeks.^22^ Healthcare resource use was recorded via self-completed diaries provided to the patient, returned at 10 and 26 weeks. Whether a new medicine had been stopped or changed was recorded by the researcher conducting the telephone interview at 10 and 26 weeks.

### Data collection

Research staff who interacted with patients were trained in minimising patient attrition. The NMS-trained pharmacist in the pharmacy consented patients into the study. Patients were called by the research team up to seven times, inside and outside working hours. Prepaid envelopes were enclosed for return of data collection items. Return-to-sender stickers were attached to all outgoing mails. Where data were not returned before a scheduled telephone call, the researcher offered to collect these data over the phone. A final request to return any study questionnaires or diaries was made at week 26.

### Statistical analysis

Complete case and intention-to-treat (ITT) analysis was applied in the same way for the 26-week data as for the 10-week analysis.^7 23 24^ Adherence rates were analysed using the χ^2^ test or Fisher’s exact test. To assess the association between non-adherence and treatment group and also to account for important potential confounders (recruiting pharmacy, age, sex, disease, medication count) the following ITT analyses were completed:

- ‘Naïve’ results: (Model 1) simple logistic regression analysis to assess unadjusted effect of NMS on the outcome.
- Main analysis: (Model 2) logistic regression analysis, adjusting for factors that, *ex ante*, are expected to influence effect size.
- Missing data on outcome: (Model 3) multiple imputation by chained equations analysis of model 2, to deal with missing data.

Models were derived for the primary outcome and for MMAS-8. Statistical analysis was conducted using Statistical Package for the Social Sciences (SPSS) V.20^25^ and Stata V.13.0.^26^

Patients who reported adherence status (measured using the NMS question) at both 10 and 26 weeks were analysed separately for both treatment arms to explore changes in adherence using McNemar’s test.

### NHS costs

Resource use data up to 26-week follow-up were collected via patient diaries. These data were combined with NHS reference^27^ and Personal Social Services Research Unit^28^ unit costs to derive total costs (online supplementary appendix 3). The cost of providing the NMS intervention was added to this total. Comparisons between treatment arms at patient level were made using a two-sample t-test on the original data set, or on a bootstrapped data set, depending on the normality of the distribution of costs.^29^

### Economic analysis

Trial design precluded observation of long-term outcomes and costs from changes in adherence. Many benefits of improved adherence are delivered well into the future. The cost-effectiveness of the NMS compared with normal practice was updated using 26-week adherence results. We simulated the effect of observed adherence increases on longer term patient outcomes (expressed as QALYs) and net NHS costs, following published reporting and model validation criteria.^30 31^ We combined the results from the NMS trial with disease-specific models to generate estimates of patient outcomes and NHS costs, over a lifetime horizon, from an NHS perspective, weighted for the proportion of NMS episodes in each disease area (online supplementary appendix 4).

The detailed methods have been published as part of the economic analysis using 10-week effectiveness data.^8^ This updated analysis reports the impact of the 26-week effectiveness data on estimates of cost-effectiveness without changing any other parameters or model assumptions, using the UK Treasury-recommended 3.5% discount rate for both costs and outcomes, using cost year 2014.

Six Markov models were developed in TreeAge Pro (TreeAge Software, Williamstown, MA, USA). The most commonly prescribed medicine within the NMS areas was used to inform a model representative of that disease group. For more details, see online supplementary appendix 5. Each model described the consequences of being adherent to the medicine, compared with non-adherence. We were able to estimate, over a lifetime, how many QALYs would be generated, and level of NHS costs incurred, in a person who was adherent, or non-adherent, to their medicine. Summaries of probabilities, costs and utilities in the Markov models are provided in online supplementary appendix 5.

Incremental costs and outcomes associated with each disease were incorporated additively into a composite probabilistic model and combined with adherence rates and intervention costs from the trial. Costs per QALY were calculated from the perspective of NHS England.

Deterministic and probabilistic incremental economic analyses were carried out. The incremental cost per QALY generated by NMS over normal practice was calculated using the following equation:

$$\left(Cost_{NMS}–Cost_{Normalpractice})/(QALY_{NMS}–QALY_{Normalpractice}\right)$$

Using Microsoft Excel, we used 5000 Monte Carlo simulations to obtain the incremental cost-effectiveness ratio (ICER) distribution. Negative ICERs are difficult to interpret and often arise when one of the interventions is either ‘dominant’ (more effective, less costly) or ‘dominated’ (less effective, more costly). It is not possible to tell this from the ICER itself. Cost-effectiveness acceptability curves^32^ were constructed to express the probability that NMS is cost-effective as a function of the decision-maker’s ceiling cost-effectiveness ratio (λ).^33^

## Results

Between July 2012 and September 2013, a total of 504 patients were recruited from 46 pharmacies (range 1–99 patients). (See online supplementary appendix 1 for CONSORT diagram.) The two groups had similar patient characteristics (table 1).

**Table 1:** Patient characteristics

Patient characteristics	Normal practice n (%)	New Medicine Service n (%)
Total n (%)	253 (100.0)	251 (100.0)
Antiplatelet/anticoagulant (n=43, 8.5%)	19 (7.5)	24 (9.6)
Asthma/COPD (n=117, 23.2%)	58 (22.9)	59 (23.5)
Hypertension (n=249, 49.4%)	128 (50.6)	121 (48.2)
Type 2 diabetes (n=95, 18.8%)	48 (19.0)	47 (18.7)
Female (n=260, 51.6%)	135 (53.4)	125 (49.8)
Male (n=244, 48.4%)	118 (46.6)	126 (50.2)
Total cohort age (years) (n: mean (SD))	253: 59.3 (15.0)	251: 59.5 (15.3)
Female age (years) (n: mean (SD))	135: 58.7 (15.4)	125: 56.8 (16.0)
Male age (years) (n: mean (SD))	118: 60.0 (14.6)	126: 62.2 (14.1)
Number of NMS eligible new medicine(s) at study entry, n (%)	Total NMS medicines: 257	Total NMS medicines: 262
1	249 (98.4)	241 (96.0)
2	4 (1.6)	9 (3.6)
3	0 (0.0)	1 (0.4)
Mean (SD) number of other medicines	3.6 (3.4)	3.5 (3.4)
Economic deprivation based on IMD score* (mean (SD))		
Pharmacy study sites	30.7 (14.0)	31.1 (13.6)
Study patients	25.0 (15.0)	24.2 (15.3)
Location of pharmacy study site, n (%)		
Derbyshire (9 pharmacies)	46 (18.2)	55 (21.9)
South Yorkshire (5 pharmacies)	35 (13.8)	31 (12.4)
Leicestershire (8 pharmacies)	15 (5.9)	10 (4.0)
Nottinghamshire (14 pharmacies)	117 (46.2)	114 (45.4)
Greater London (10 pharmacies)	40 (15.8)	41 (16.3)
Pharmacy ownership†, n (%)		
Independent	65 (25.7)	56 (22.3)
Large multiple	63 (24.9)	68 (29.1)
Small multiple	122 (48.2)	123 (49.0)
Supermarket	3 (1.2)	4 (1.6)

*Index of Multiple Deprivation (IMD) score: score is proportional to level of deprivation. A higher score indicates an area of higher deprivation (English deprivation scores range from 0.5 to 87.8).

†Large multiple and supermarket: the 10 largest pharmacy entities in England. Small multiple: pharmacies with six or more branches. Independent: pharmacies with one to five branches.

COPD, chronic obstructive pulmonary disease; NMS, New Medicine Service.

At 26 weeks, 41 and 25 patients had withdrawn from the normal practice and NMS arms, respectively. Reasons given for withdrawal were (normal practice, NMS arm): deceased (1, 0), not having time for study (6, 1), health reasons (3, 3), no longer interested in study (3, 3), no longer taking new medicine (2, 1), no specific reason stated (13, 11), patient had not started new medicine (2, 0), personal reasons or circumstances (2, 4), study design (2, 0), withdrawal due to recruitment issues (3, 1), patient had medicine before (0, 1) and withdrawal at randomisation (4, 0). At 26-week follow-up, 362/438 (83%) phone calls were successful, 245/440 (56%) of 26-week questionnaires were returned and 209/439 (48%) of 6-month diaries were returned.

### Effect of NMS on adherence at 26 weeks

Results for adherence, measured with the NMS question and MMAS-8 at week 26 follow-up, are shown in table 2. For comparison, we show the data from week 10 which has previously been reported.^7^ All the analyses met statistical significance at week 10, but none achieved this at week 26.

**Table 2:** Reported adherence by patients to their new medicine (measured using the NMS question and Morisky Eight-Item Medication Adherence Scale (MMAS-8) and intention-to-treat analysis of the intervention as a predictor of adherence at weeks 10 and 26—frequency counts, unadjusted, adjusted and imputed ORs

Intention-to-treat analysis at week 10 or 26 follow-up	Adherent patients/total responses, n (%), P value	Model 1* (naïve) OR (95% CI, P value)	Model 2 (main/adjusted) OR (95% CI, P value)	Model 3 (imputation) OR (95% CI, P value)
**10-week follow-up: adherence as assessed by NMS question (n=378**)				
Normal practice	115/190 (60.5)	1.00	1.00	1.00
NMS intervention	133/188 (70.7), 0.037	1.58 (1.03 to 2.42, 0.037)	1.67 (1.06 to 2.62, 0.027)	1.62 (1.04 to 2.53, 0.032)
**26-week follow-up: adherence as assessed by NMS question (n=327**)				
Normal practice	97/170 (57.1)	1.00	1.00	1.00
NMS intervention	103/157 (65.6), 0.113	1.44 (0.92 to 2.25, 0.114)	1.50 (0.93 to 2.44, 0.095)	1.50 (0.89 to 2.51, 0.127)
**Secondary outcomes (adherence as assessed by MMAS-8**)				
**10-week follow-up adherence MMAS-8 (n=267**)				
Normal practice	85/143 (59.4)	1.00	1.00	1.00
NMS intervention	89/124 (71.8), 0.035	1.74 (1.04 to 2.90, 0.036)	1.88 (1.06 to 3.34, 0.030)	1.77 (0.96 to 3.28, 0.068)
**26-week follow-up adherence MMAS-8 (n=223**)				
Normal practice	70/124 (56.5)	1.00	1.00	1.00
NMS intervention	63/99 (63.6), 0.277	1.35 (0.79 to 2.32, 0.278)	1.60 (0.84 to 3.06, 0.153)	1.43 (0.70 to 2.91, 0.320)

*Model 1 (naïve): simple logistic regression model. Model 2 (main/adjusted): logistic regression model adjusted for recruiting pharmacy, disease, age, sex and medication count. Model 3 (imputation): adjusted logistic regression model taking imputation of missing data into account.

NMS, New Medicine Service.

In the unadjusted complete case analysis of 327 patients still taking the initial medicine at 26 weeks, 97/170 (57.1%) and 103/157 (65.6%) (p=0.113) patients were adherent in the normal practice and NMS arms, respectively. In the main analysis (model 2), adherence yielded an OR of 1.50 (95% CI 0.93 to 2.44, p=0.095), in favour of the NMS arm.

Marginal effects were estimated as probabilities for models 1 and 2 for primary and secondary outcomes at 26-week follow-up and are reported in the online supplementary appendix 6. The method applied was marginal probabilities at the mean, given that all patients were in one or the other arm, respectively. These results suggest that, when using the NMS question to measure adherence, 69% of patients in the NMS arm were adherent to their medicine at 26 weeks, compared with 60% in the normal practice arm, an absolute difference of 9.0%, but this difference is not statistically significant.

Three hundred and five patients responded by telephone at both 10 and 26 weeks, respectively, 191 patients returning questionnaires for both time points. Adherence for patients providing data at both time points shows little variation by arm or adherence measure in the overall cohort (table 3A). At the cohort level, reported changes in adherence behaviour (non-adherent patients becoming adherent or adherent patients becoming non-adherent) were similar in both arms (table 3B). In the NMS intervention arm, of 44 non-adherent patients, 17 (38.6%) become adherent between 10 and 26 weeks. Of 102 adherent patients, 19 (18.6%) become non-adherent, with no significant change in adherence between 10 and 26 weeks for the overall cohort (two-sided p=0.87). In the normal practice arm, of 65 non-adherent patients, 10 (15.4%) become adherent. Of 94 adherent patients, 10 (10.6%) become non-adherent, with no significant change in adherence between 10 and 26 weeks for the overall cohort (two-sided p=1.0).

**Table 3A:** Reported adherence by patients to their new medicine (measured using the NMS question and Morisky Eight-Item Medication Adherence Scale (MMAS-8)) for patients providing adherence data at both 10 and 26 weeks

	Adherent patients/total responses, n (%)			
	NMS question (n=305)		MMAS-8 (n=191)	
	10 weeks	26 weeks	10 weeks	26 weeks
Normal practice	94/159 (59.1)	94/159 (59.1)	63/112 (56.3)	62/112 (55.4)
NMS intervention	102/146 (69.9)	100/146 (68.5)	57/79 (72.2)	55/79 (69.6)

NMS, New Medicine Service

**Table 3B:** Reported adherence by patients to their new medicine (measured using the NMS question) showing response combinations for patients providing adherence data at both 10 and 26-week time points (n=305)

		26-week response (n)			
		Normal practice		NMS intervention	
		Non-adherent	Adherent	Non-adherent	Adherent
10-week response (n)	Non-adherent	55	10	27	17
	Adherent	10	84	19	83
OR (95% CI; two-sided p value)*		1.00 (0.37 to 2.68; 1.0)		0.89 (0.44 to 1.82; 0.87)	

*Repeated measures McNemar’s test, OR>1 indicates improved adherence from 10 to 26 weeks, OR=1 no change, OR<1 deteriorated adherence.

NMS, New Medicine Service

By week 26, across both groups, there were 60 (13.7%) reports of patients whose medicines had been changed and 52 (11.9%) reports of patients with medicines stopped by their clinician. The medicine most frequently changed or stopped was amlodipine.

No reports of patient harm were reported to the study team by the patient, pharmacy team members, prescriber or researchers as a result of the intervention or participation in the study.

### Effect of NMS on NHS costs at 26-week follow-up

Mean (n, median, range, SD) total NHS costs for patients in normal practice and NMS arms are £520.21 (126, £244.97, £0–£4188.73, £62.04) and £415.84 (132, £212.02, £0–3384.06, £46.45), respectively. There was a general trend to reduced NHS costs, although statistically non-significant, for the NMS intervention: −£104.36 (95% CI £37.84 to −£256.52, p=0.168).

### Economic analysis

**Figure 1:**
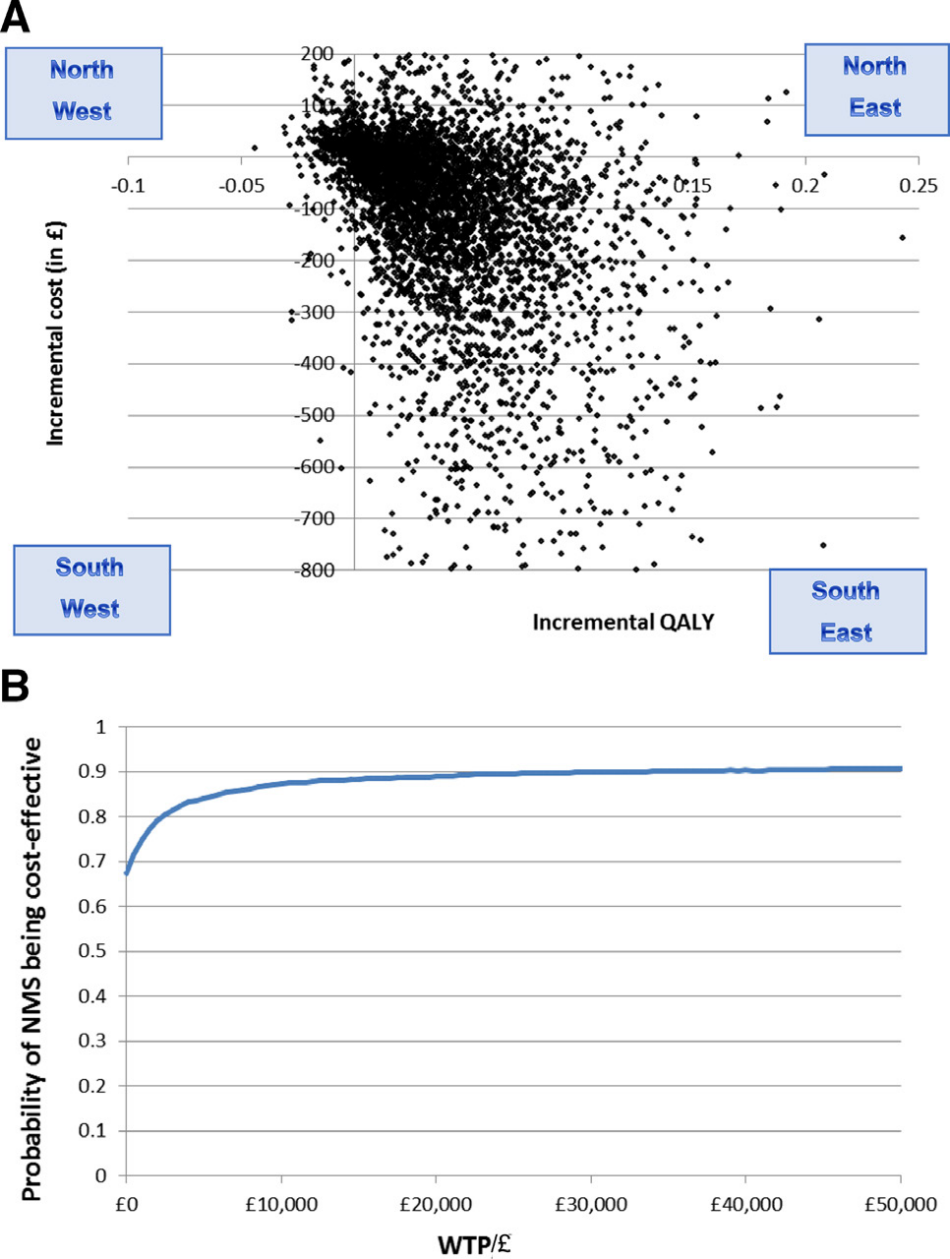
(A) Incremental cost-effectiveness plane: NMS intervention versus current practice. (B) Cost-effectiveness acceptability curve (NMS intervention vs current practice). This graph demonstrates the probability of cost-effectiveness at a range of decision-maker ceiling willingness-to-pay values for the NMS intervention overall. NMS, New Medicine Service; QALY, quality-adjusted life-year; WTP, willingness to pay.

The probabilistic analysis reported that NMS generated a mean of 0.04 (95% CI −0.01 to 0.13) more QALYs per patient, at a mean reduced cost of −£113.9 (95% CI −1159.4 to 683.7) (see table 4). The deterministic analysis reported very similar results (online supplementary appendix 4). The NMS dominates normal practice with probability of 0.653 (ICER: −£2847.5 per QALY, 2.5%, 97.5% percentiles: −38 739.5, 34 024.2) (see figure 1A). NMS has an 89.0% probability of cost-effectiveness compared with normal practice at a willingness to pay of £20 000 per QALY (see figure 1B).

**Table 4:** Incremental economic analysis of NMS versus normal practice at 26-week follow-up

Mean cost (2.5%, 97.5% percentiles)/£		Mean QALY (2.5%, 97.5% percentiles)		Incremental difference (2.5%, 97.5% percentiles)		ICER: £/QALY (2.5%, 97.5% percentiles)
NMS*	Normal practice	NMS	Normal practice	Cost/£	QALY	
20 482.7 (9438.9, 53 822.0)	20 596.5 (9435.5, 54 125.5)	13.45 (12.55, 14.35)	13.41 (12.50, 14.31)	−113.9 (−1159.4, 683.7)	0.04 (−0.01, 0.13)	−2847.5 (−38 739.5, 34 024.2)

*Incorporating cost of intervention equal to £24.6.

ICER, incremental cost-effectiveness ratio; NMS, New Medicine Service; QALY, quality-adjusted life-year.

## Discussion

At week 26, the NMS increased the proportion of patients reporting adherence to their new medicine by 8.5% (65.6%, compared with the 57.1% adherence in normal practice, p=0.113). The adjusted effect size was 9.0% (p=0.095). This effect was of smaller magnitude than at week 10: 10.2%, adjusted effect size 11.0%.^7^ This loss of statistical significance could be due to loss of effect of the intervention. A reduced sample size (51 fewer patients provided information), resulting in widened CIs, could also have had some influence.

At the study population level there was little difference between 10-week and 26-week findings. Patient-level analysis suggests that there is mobility of patients between states of adherence, however this appears broadly even in both directions producing a net zero change, suggesting that at patient level, adherence is a dynamic behaviour over time, but, at population level, there is some degree of persistence of effect of NMS over time.

There is a bigger reduction in NHS costs at 26 weeks (£104) than at 10 weeks (£21),^7^ but still not statistically significant. This could be due to a combination of loss of effect of NMS, patient attrition and high variability in patient costs. This bigger reduction could be attributed to the increased proportion of patients adhering to their medicines in the NMS arm having better disease control for 26 weeks leading to continued reduced use of (mostly secondary care) NHS resources. These results strengthen our earlier conclusions that the short-term cost of NMS to the NHS is absorbed by reduced NHS costs.

The economic evaluation suggests NMS will deliver better patient outcomes, measured as QALYs, than normal practice at overall reduced costs to the NHS in the long term. At 26 weeks, NMS has an 89.0% probability of cost-effectiveness compared with normal practice at a willingness to pay of £20 000 per QALY, which is the threshold for cost-effectiveness in the NHS in England. This is despite the loss of statistical significance of the effect size at 26 weeks contributing to increased uncertainty around ICER point estimates.

### Strengths and limitations

Key strengths of this study are the rigorous design, pragmatic nature, economic analysis, long-term follow-up, and extensive observational and interview-based work alongside the RCT to examine implementation from the perspectives of patients, pharmacy teams and prescribers.^34–36^

The study was powered for the evaluation of effectiveness at 10 weeks, with 26 weeks being designed, a priori, as a secondary outcome. This priority was agreed with the Department of Health, which funded the study. We were also aware that so little was known about the possible future outcomes at 26 weeks that there would have been significant uncertainty in any sample size estimation. Unfortunately, there was insufficient funding to conduct the 26-week analysis and the research team dispersed, leading to the delay between this paper and the presentation of the 10-week adherence data in 2016 and economic analysis in 2017.

Sixty-six patients withdrew between weeks 10 and 26, 62% from the normal practice arm; the CIs increased, the effect size diminished and did not meet statistical significance at the p=0.05 level. Patient attrition at 26 weeks is not surprising given the commitment and motivation needed to take part over a long period of time. Patients may feel more confident using the medicine and therefore motivation to continue with the study may have reduced. They may also forget about the study, especially those in the control arm.

A further limitation was the use of a patient-reported behavioural process measure, and the need to extrapolate from this to estimate impact on patient health and healthcare provider costs. There is no gold standard for measuring patients’ medicine adherence. We used two self-report measures to provide an internal check on validity.^37^ Although self-report tends to return a higher rate of medication adherence (+15%) than some objective measures, it correlates with objective clinical measures.^38^ We minimised biases through confidential interview,^39^ made efforts to normalise non-adherence by recognising the challenges of taking regular medications and asked about missed doses only in the week prior to data collection, to optimise recall.^40^ Some confidence is given by our use of both telephone and postal methods for reporting adherence, which yielded similar ORs.

Adherence to medicines for long-term conditions drops over time,^41–43^ so adherence was expected to drop in the normal care arm. We assumed it would drop by a similar extent in the NMS group.^8^ The results from the 26-week analysis partially support this, as the effect size reduced from 11.0% at 10 weeks to 9.0% at 26 weeks. The apparent partially sustained effect is encouraging, given effects of cognitive interventions tend to reduce over time, particularly short ‘one-off’ interventions. This may suggest that if problems are caught early there is some partial maintenance of effect, but additional work is required to examine this further.

A limitation of the economic evaluation is the paucity of published evidence upon which to base the estimates of economic impact of adherence in the individual disease–drug pairs, particularly the variety of ways in which adherence is measured in different studies, and the link between adherence and outcome. The wide range around the point estimates of cost-effectiveness reflects the lack of statistically significant difference in adherence, and the uncertainty in some of the individual adherence models.

### Implications for clinicians and policymakers

Since approval, the NMS has had wide and sustained adoption in England. To the end of December 2018, 5.718 million consultations have been claimed for in England, with over 926 000 in the year 2017/2018, and 12 036 pharmacies have claimed for at least one NMS consultation.^44^ Estimates of the benefits of NMS since its introduction, using the current economic evaluation, are £651.8 million long-term cost savings to the NHS and 228 715 QALYs gained.

To understand perception, implementation and execution of NMS in a real-world setting, our work included in-depth exploration of NMS with key stakeholders (patients, prescribers, pharmacists), providing essential insight into future developments and commissioning.^34–36^ The NMS workload had been absorbed into pharmacists’ daily routines alongside existing responsibilities with no extra resources or evidence of reduction in other responsibilities.^34^ We found pharmacists were pragmatic, simplifying and adapting the NMS to facilitate its delivery which may have affected fidelity and thus effectiveness. Pharmacist understanding of the NMS was found to impact on what they believed should be achieved from the service.^36^ Patients who had few problems with medicines had varied reactions to the service.^36^ Where there was ambiguity or poor prior understanding of the NMS, people were reluctant to engage with the service. As the service becomes more embedded in practice, and as patients become more accustomed to such interactions, it is likely that patients will be able to frame the service as intended and so potentially benefit from the support offered. A review of promotional and public campaigns is needed to generate engagement among publics who may be indifferent, medically underserved or unacquainted with newer pharmacy services.^34 36 45^ The NMS results in a more complex configuration of relational power between pharmacists, patients and prescribers.^35^ We found that GPs were generally supportive of the initiative but unaware of the service or potential benefits. The service is likely to gain greater traction if it was seen to align with prescribers’ priorities.^36^

The NMS was designed to be the first step in supporting a patient starting to use medicines to maintain a long-term condition. A very similar intervention has been demonstrated to have similar effectiveness in people established on statins or diabetic medicines.^46^ This suggests that using this approach when the medicine is started, and reviewing it every 6 months (NHS England already funds pharmacists to conduct six monthly reviews of patients on several long-term medications), could have a sustained effect. Such a service would support national policy. NHS England proposes more appropriate clinical use of community pharmacy,^47^ and has introduced clinical pharmacists into primary care doctor’s practices.^48^ It is essential to examine how NMS integrates with these primary care service developments.

## Conclusions

At 26-week follow-up, NMS no longer demonstrated a statistically significant increase in adherence or reduction in NHS costs, which may be attributable to patient attrition from the study. Long-term economic evaluation suggested NMS may deliver better patient outcomes and reduced overall healthcare costs than normal practice at 26 weeks, with findings of increased health gain with NMS over normal practice at a cost per QALY well below most accepted thresholds for technology implementation.^49^ However, uncertainty around this finding is very high, and more work is needed to understand how to sustain intervention effects over time.
