# Supplementary material for: ‘New Medicine Service’: supporting adherence in people starting a new medication for a long-term condition: 26-week follow-up of a pragmatic randomised controlled trial
Source: BMJ Qual Saf. 2019 Nov 15;29(4):286–95. doi: 10.1136/bmjqs-2018-009177 (PMC7146933; doi:10.1136/bmjqs-2018-009177)
Supplement: Supplementary data [file bmjqs-2018-009177supp001.pdf]

Support for people starting a new medication for a long-term condition through community pharmacies: a pragmatic randomised controlled trial of the New Medicine Service: Supporting information

Elliott RA, *et al. BMJ Qual Saf* 2020; 29:286–295. doi: 10.1136/bmjqs-2018-009177

Appendix 1 Pharmacy and Patient Recruitment

Pharmacy recruitment

Eligibility for pharmacies to join the trial

Community pharmacies providing NMS in East Midlands and South Yorkshire (EMSY) and Greater London (GL) were eligible to take part in the study. Pharmacy selection took into account known variables that influence organisational structures, work flow and integration, including ownership, proximity to GP, setting and economic deprivation.

Ownership: We used the categorisation of pharmacy ownership based on the criteria used by PricewaterhouseCoopers (PwC) in their cost of service analysis of pharmacy for the DH.[1] It is understood that these definitions were agreed by the DH and by PSNC. PwC suggested that large multiples (Boots, Lloyds, Rowlands, Co-op, Superdrug and Day Lewis) and supermarkets (Sainsbury's, Morrisons, ASDA and Tesco) were defined as the ten largest pharmacy entities in England. Of the remaining entities, independents were defined as those with one to five branches and smaller multiples as those with six or more branches.[1] PwC report that there are 3890, 997, and 5310 pharmacies for each of independents, small multiples and large multiples (inc supermarkets) a total of 10197 giving relative proportions of 37.8%, 9.7% and 52.1% respectively. According to the Office of Fair Trading (OFT) large multiples represented 24.5% of outlets, supermarkets, 6.8% of outlets, small multiples 6.5% and independents 42.1%. This represents the figures on 1st October 2009.[2]

These numbers from the PwC and OFT report represent the number of pharmacies providing NHS services, not those providing the NMS. Data from the PSNC (http://psnc.org. uk/funding-and-statistics/nhs-statistics/nms-statistics/) suggest that on average, between 6000-7000 pharmacies of the approximately 10 500 pharmacies claim for at least one complete NMS per month. To March 2013, 10 101 pharmacies had claimed for at least one NMS consultation.

The proportions in the OFT report were used as a starting point for working proportions of pharmacies to recruit by ownership type in order to represent the national picture. In the absence of any intelligence on NMS figures we had to pick a surrogate distribution, the only real option available being ownership. We used OFT reported proportions as they separated out supermarkets from other large multiples and we felt NMS may be different in these two categories. However, in the RCT, pharmacies delivering the NMS were selected, and then we had to select those recruiting patients at a minimum of a notional rate of two or more per week, so we did not necessarily expect the ownership proportions of our sample to match those of the OFT. For reasons of commercial sensitivity, PharmOutcomes were unable to provide details of which pharmacy delivered each NMS in the national dataset so we are unable to compare our sample with that of NMS conducted across England.

Proximity to GP: over 90% of pharmacies are within 500m of a GP surgery, a further 5% are between 500m and 1km and the remainder are over 1km away. About 30% of pharmacies are co-located.[2] The average (mean) prescription is written 132m away from a community pharmacy.

For this study the proximity of pharmacy to GP was taken as the line of sight distance from pharmacy site to nearest GP surgery by postcode, using the NHS Choice website (http://www.nhs.uk/Service-Search/GP/ LocationSearch/4).

Elliott RA, *et al. BMJ Qual Saf* 2020; 29:286–295. doi: 10.1136/bmjqs-2018-009177

Setting: The setting was on the basis of urban, sub-urban and rural categories. In 2004 the Department for Environment Food and Rural Affairs came up with a consensus of the terminology to be used across government.[3] This classified each output area (government breakdown of the country) into areas based on morphology and context. The morphology is separated into: urban (population over 10,000), rural town, village and dispersed (hamlets and isolated dwellings); and context as sparse or less sparse. This in total gives eight urban/rural classifications. The setting for each pharmacy was established using data from the Office for National Statistics using the pharmacy's postcode as the lookup reference.[4]

Economic deprivation: The UK Office for National Statistics (ONS) provide publicly available data that describes each area of the England in terms of its deprivation, the Index of Multiple Deprivation (IMD). Two measures are provided for each area one is a score based on a series of deprivation indicators, the higher the score the higher the deprivation of an area. These scores are then ranked across England to show relative deprivation, the area with a deprivation rank of one being the most deprived area of England. For this study the latest ONS data available was used.[5] Data from the Office of National Statistics was also used to ascertain the deprivation index for each pharmacy using the postcode as the lookup reference.[6] Data were collected for two variables: (i) Index of Multiple Deprivation (IMD) Score and (ii) rank of IMD score. The IMD score is directly proportional to the level of deprivation (higher IMD score; higher level of deprivation) while the IMD rank is inversely proportional to the level of deprivation (lower IMD rank; higher level of deprivation). The Office of National Statistics data records the English deprivation scores as ranging from 0.5 to 87.8 and deprivation rank scores ranging from 1 to 32482.

Recruitment of community pharmacies

Phase one recruitment:

Pharmacies were recruited into the trial using a pragmatic convenience sample to enable a representative sample across the four eligibility criteria (ownership, proximity to the GP, setting, and economic deprivation). Assistance in finding suitable pharmacy sites was sought from Local pharmaceutical committees (LPCs) and other regional and national pharmacy bodies in the GL and EMSY areas. Superintendent pharmacists of a range of multiple-owned pharmacy organisations were also approached for and permission sought for a sample of their pharmacies to be included. Phase one recruitment included an early pilot consisting of four pharmacy sites to ensure that training, setup of the pharmacy to operationalise the study, recruitment methods, study materials and processes were satisfactory before full roll out to all phase one pharmacies. Four patients were recruited as part of the pilot prior to wider roll out. Data collected for these patients were included in the final analysis.

Phase two recruitment:

Challenges with patient recruitment due to lack of both NMS service uptake and patients declining to be in the study lead to a second phase of pharmacy recruitment commencing in April 2013. The main focus of this recruitment phase was to supplement the pharmacies that had not recruited any patients with 'NMS-active' pharmacies. We had recruited 24 pharmacies, exceeding our target of 22, but at least ten had failed to recruit any patients by this point. Those pharmacies that were more successful at recruiting patients to the RCT were identified and investigated to understand the characteristics that made them successful recruiters. Data gathered from pharmacy profiling was used to develop a pharmacy recruitment tool to enable identification of future sites that would be potentially more successful at recruiting patients to the study. A 'site suitability survey' was developed and used to inform future site recruitment.[7] The PSNC and primary care research network also provided support in identifying 'NMS-active' pharmacies.

Elliott RA, *et al. BMJ Qual Saf* 2020; 29:286–295. doi: 10.1136/bmjqs-2018-009177

Randomisation and blinding

All patients were recruited by study pharmacists within community pharmacies. Pharmacists were instructed to invite patients to the study only after they had consented to accept the NMS. Patients, and where applicable their representative, were provided with a succinct and comprehensive communication about what was involved in the study by a study-trained pharmacy team member. The patient was encouraged to read the study documentation within the privacy of the consultation area and to ask questions about the study before considering participation. The study-designated pharmacist was responsible for checking the patients' understanding of the study, answering patients' questions and for consenting the patient into the study. Once the patient had consented, the study-designated pharmacist opened a sealed tamper-proof envelope to reveal what arm of the study the patient had been randomised to. Consent to continue in the study was reconfirmed when patients were contacted by our research team. Study posters were clearly displayed in each pharmacy, allowing the option for patients to volunteer themselves for the study. At all stages of the process the patient was assured that study participation was entirely optional and their decision to take part or not to take part in the study would not have an impact any care they receive,

Patients were randomised into one of the two study arms stratified by drug/disease group within each pharmacy using Statistical Analysis Software.[8] Block randomisation was used within each pharmacy to avoid allocation imbalances. Sequentially numbered tamper-proof opaque sealed envelopes were used to conceal sequence allocation. Separate randomisation sequences were produced for patients 16 years and over and for patients aged 14 years and 15 years, due to the age-specific motivators for adherence in this latter group.[9] Researchers collecting data were blinded to study arm except in the case of accidental disclosure by study participants or when inviting a participant to the qualitative arm of the study.

Pharmacists were not given any steer on what patients to include in the trial, other than those they would normally recruit, nor target numbers.

Pharmacies were asked to record patients approached who declined to participate in the study. Of the 369 recording periods over the 61 pharmacies, data were received for 94 of these periods. In the remaining periods either proformas were not returned or returned not completed. 470 declines were recorded across the 94 periods. Declines by pharmacy ranged from 1 to 150.

Elliott RA, *et al. BMJ Qual Saf* 2020; 29:286–295. doi: 10.1136/bmjqs-2018-009177

Figure 1 Overview of pharmacy and patient recruitment (CONSORT) and follow-up to Week 26

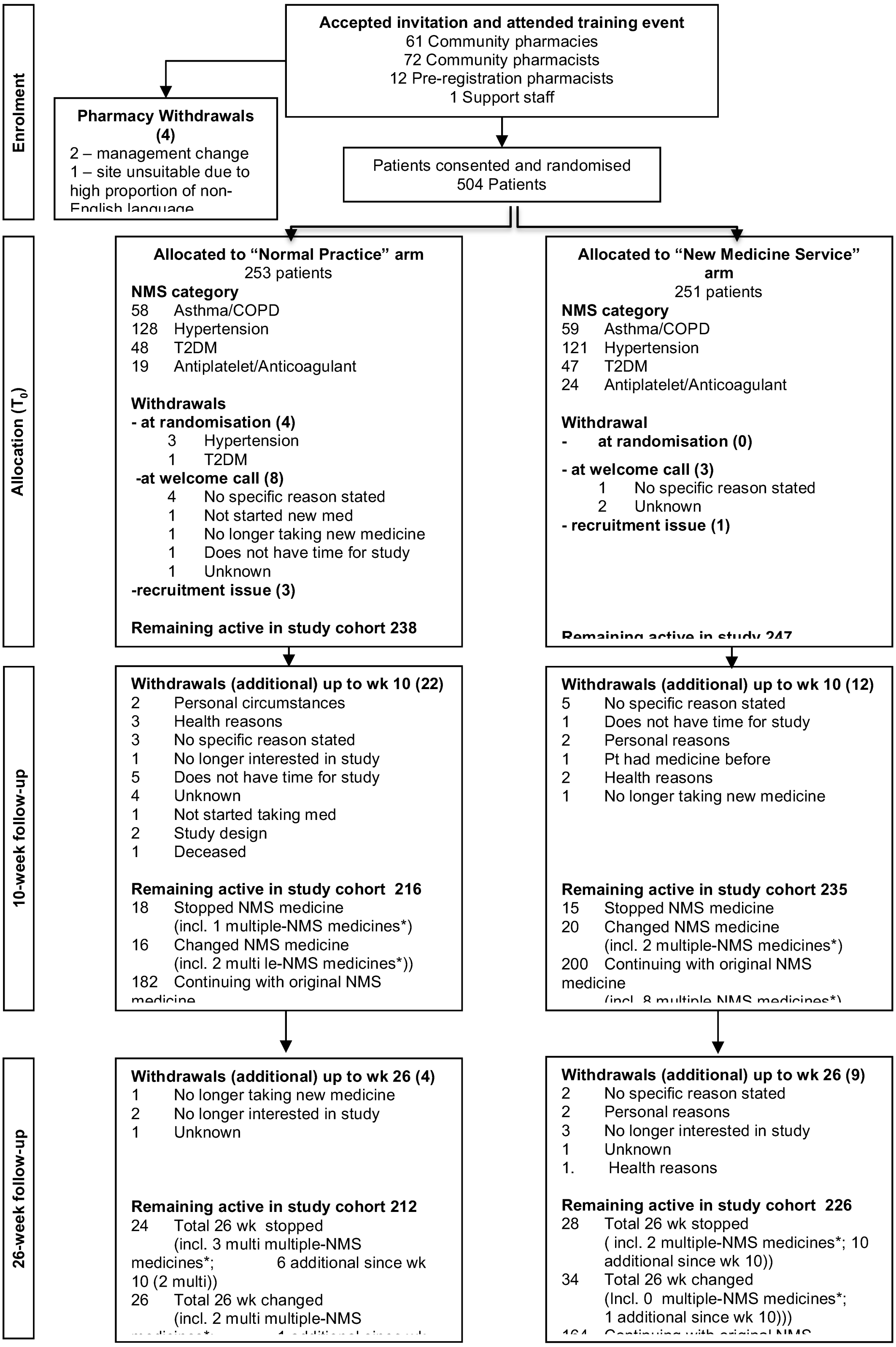

*Patient received additional NMS medicines during course of study

"Active" patients indicates how many patients are contributing data to the study at each time point, by arm.

Elliott RA, *et al. BMJ Qual Saf* 2020; 29:286–295. doi: 10.1136/bmjqs-2018-009177

Appendix 2: NMS intervention schematic diagram

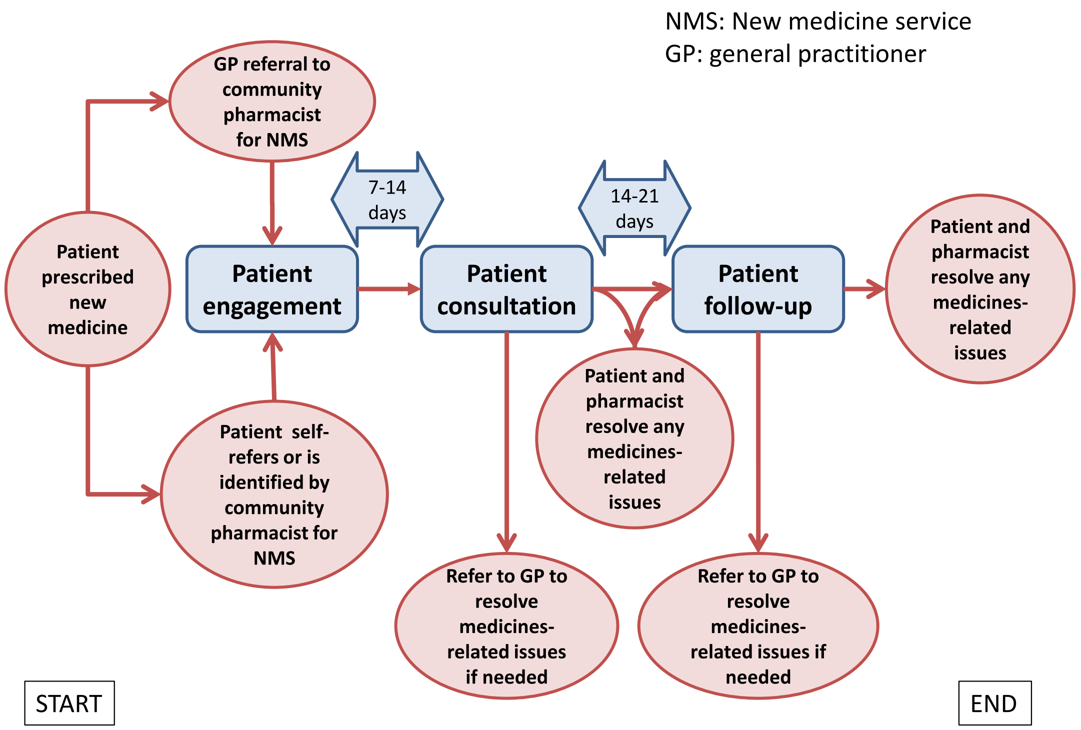

Elliott RA, *et al. BMJ Qual Saf* 2020; 29:286–295. doi: 10.1136/bmjqs-2018-009177

Appendix 3: economics and costing

Table 1 Unit costs for resource use

Cost type	Time assumptions	Cost	Source
**Primary care**			
GP admin	5 min	£14.50	[10]
GP phone call	7.1 min	£20.00	[10] table 10.8b p 191
GP home visit	23.4 min	£85.00	[10] table 10.8b p 191
GP contact	11.7 min	£34.00	[10] table 10.8b p 191
Nurse phone call	7.1 min	£4.02	[10] section 14.4, p236
Nurse home visit	27 min	£15.30	[10] section 14.4, p236, [11]
Nurse contact	15.5 min	£8.78	[10]section 14.4, p236
**Secondary care**			
Day case	-	£697.00	[10] section 7.1 p 107
Outpatient visit	-	£135.00	[10] section 7.1 p 107
Inpatient stay	-	£3,283.00	[10] section 7.1 p 107
**Allied health professionals/pharmacists***			
AHP contact	15.5 min	£10.59	[10] section 11.5 , p 201
Pharmacist contact	5.83 min	£4.96	[10] section 9.6 p.180, [12]
AHP home visit	27 min	£18.45	[10] section 11.5 , p 201 , [11] p.164
AHP phone call	7.1 min	£4.85	[10] section 11.5 , p 201
**Social care**			
Home visit	25.07 min	£66.44	[10] section 11.2 p 198, [13]
Phone contact	7.1 min	£4.73	[10] section 11.2 p 198
Contact with social care/health worker	25.07 min	£16.71	[10] section 11.2 p 198, [13]

GP: general practitioner
*AHP: podiatrists, phlebotomists

Elliott RA, *et al. BMJ Qual Saf* 2020; 29:286–295. doi: 10.1136/bmjqs-2018-009177

Table 2 Unit costs for resource use in secondary care based on the descriptions from the resource use diaries.

Cost type	Category	Description of Currency code*	Unit cost[14]
Outpatient	Ophthalmology		£85.90
Outpatient	BZ04B	Lens Capsulotomy, with CC Score	£262.68
Outpatient	Respiratory Medicine		£150.23
Outpatient	Physiotherapy		£42.47
Outpatient	Trauma and Orthopaedics		£109.65
Day case	Transient Ischaemic Attack		£206.38
Day case	Diagnostic Imaging		£37.06
Day case	BZ02C	Phacoemulsification Cataract Extraction and Lens Implant, with CC Score -1	£865.82
Day case	Cardiology		£131.41
Day case	RA60A	Simple Echocardiogram, 19 years and over	£74.96
Day case	Diabetic Medicine		£136.13
Outpatient	Anticoagulant Service		£24.59
Outpatient	Hepatology		£212.99
Outpatient	Clinical Haematology		£150.62
Outpatient	Maxillo-Facial Surgery		£110.02
Outpatient	Geriatric Medicine		£204.19
Outpatient	Cardiac Surgery		£298.74
Outpatient	Vascular Surgery		£142.40
Outpatient	Gastroenterology		£137.02
Day case	CZ08Y	Minor Ear Procedures, 19 years and over without CC	£740.72
Day case	FZ51Z	Diagnostic Colonoscopy, 19 years and over	£485.95
Day case	DZ50Z	Respiratory Sleep Study	£511.68
Outpatient	Rheumatology		£139.66
Outpatient	Nephrology		£157.69
Day case	General Surgery		£128.20
Day case	BZ03B	Non-Phacoemulsification Cataract Surgery, with CC Score	£981.66
Day case	BZ24G	Non-Surgical Ophthalmology, without Interventions, with CC Score -1	£363.46
Outpatient	Breast Surgery		£138.11
Outpatient	Neurology		£175.75
Outpatient	Cardiology		£131.41
Day case	EA45Z	Complex Echocardiogram, including Congenital, Transoesophageal and Foetal Echocardiography	£718.96
Outpatient	Chemical Pathology		£63.52
Outpatient	Diabetic Medicine		£136.13
Outpatient	Accident & Emergency		£116.88
Day case	Interventional Radiology		£263.56
Outpatient	General Surgery		£128.20
Day case	HA35Z	Minor Foot Procedures for Trauma, Category 1	£1,765.81
Day case	BZ04B	Lens Capsulotomy, with CC Score	£262.68
Day case	Clinical Haematology		£150.62
Outpatient	Colorectal Surgery		£112.69
Day case	Gynaecology		£129.81
Day case	Rheumatology		£139.66
Outpatient	Endocrinology		£151.95

Elliott RA, *et al. BMJ Qual Saf* 2020; 29:286–295. doi: 10.1136/bmjqs-2018-009177

Cost type	Category	Description of Currency code*	Unit cost[14]
Day case	FZ53Z	Therapeutic Colonoscopy, 19 years and over	£541.81
Day case	FZ52Z	Diagnostic Colonoscopy with Biopsy, 19 years and over	£554.48
Outpatient	Audiology		£70.04
Day case	Ophthalmology		£85.90
Day case	Accident & Emergency		£116.88
Day case	Trauma and Orthopaedics		£109.65
Day case	HA59Z	Minimal Hand Procedures for Trauma, with length of stay 1 day or less	£744.60
Day case	Ear nose and throat		£93.93
Outpatient	Dermatology		£97.96
Day case	Dermatology		£97.96
Outpatient	Medical Oncology		£137.58
Outpatient	Stroke Medicine		£199.56
Outpatient	Medical Ophthalmology		£92.78
Outpatient	Transient Ischaemic Attack		£206.38
Outpatient	General Medicine		£153.33
Day case	HA79Z	Minimal Elbow and Lower Arm Procedures for Trauma, with length of stay 1 day or less	£653.58
Day case	Endocrinology		£151.95
Outpatient	Urology		£101.15
Day case	FZ42A	Wireless Capsule Endoscopy, 19 years and over	£687.67
Day case	Gastroenterology		£137.02
	Diagnostic Imaging		£37.06
Outpatient	Interventional Radiology		£263.56
Outpatient	Pain Management		£138.17
Day case	HB12C	Major Hip Procedures for Non-Trauma, Category 1, without CC	£2,524.89
Outpatient	Upper Gastrointestinal Surgery		£119.68
Outpatient	Adult Mental Illness		£221.49
Outpatient	Ear nose and throat		£93.93
Day case	AA29D	Transient Ischaemic Attack with CC Score 8-1	£782.47
Day case	HA93Z	Foot Trauma Diagnosis without Procedure	£678.78
Outpatient	Gynaecological Oncology		£137.73
Day case	Neurology		£175.75
Outpatient	Cardiac Rehabilitation		£42.25
	General Medicine		£153.33
Outpatient	Genitourinary Medicine		£115.31
Outpatient	Gynaecology		£129.81
Day case	EB04Z	Hypertension	£463.66
Outpatient	Dietetics		£64.20
Day case	AA35F	Stroke with CC Score -3	£520.14
Outpatient	Occupational Therapy		£63.10
Day case	Respiratory Medicine		£150.23
Day case	Urology		£101.15
Day case	FZ17G	Abdominal Hernia Procedures, 19 years and over with CC Score	£1,361.26
Day case	JC43A	Minor Skin Procedures, 13 years and over	£623.84

Elliott RA, *et al. BMJ Qual Saf* 2020; 29:286–295. doi: 10.1136/bmjqs-2018-009177

Cost type	Category	Description of Currency code*	Unit cost[14]
Inpatient	EA19C	Excess bed day cost	£1,915.59
Outpatient	Obstetrics		£122.35
Inpatient	NZ50C	Planned Caesarean Section, with CC Score -1	£1,353.08
Outpatient	Podiatry		£42.16
Outpatient	Accident & Emergency		£116.88
Outpatient	Respiratory Physiology		£119.22
Outpatient	Liaison Psychiatry		£107.69

CC: concomitant comorbidities

*Description of 5 digit currency code as appeared on the NHS reference schedule

Elliott RA, *et al. BMJ Qual Saf* 2020; 29:286–295. doi: 10.1136/bmjqs-2018-009177

Appendix 4: Overview of economic model developed to combine NMS trial results with estimates of harm caused by non-adherence

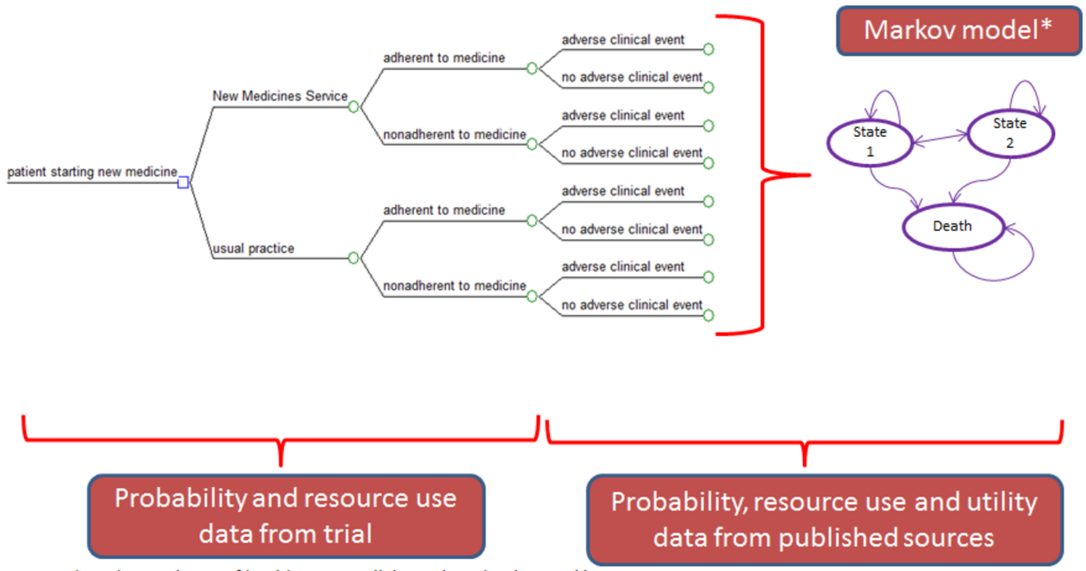

*number and type of health states will depend on the disease/drug group

Elliott RA, *et al. BMJ Qual Saf* 2020; 29:286–295. doi: 10.1136/bmjqs-2018-009177

Appendix 5: Economic analysis: sources of models

Asthma and COPD were modelled separately due to the different natural history of the disease and impact of non-adherence. As hypertension represented over 50% of the cohort, two models were built to reflect the two most common medication groups prescribed: calcium channel blockers (CCB) and angiotensin-converting enzyme (ACE) inhibitors—index NMS drugs for 34.4% and 24.1% of hypertensive patients, respectively. Where available, we utilised and adapted existing published models to optimise design: hypertension-CCB (amlodipine)[15], hypertension-ACEI (ramipril)[16], asthma (beclometasone)[17], COPD (tiotropium)[18], type 2 diabetes (metformin).[19] We derived the antiplatelet model structure from case-control studies analysing outcomes in large UK cohorts of patients with a first prescription of aspirin.[20 21]

Economic analysis: sources of data to populate models

Clinical event probability, treatment pathway, resource-use and cost data were extracted from literature and costing tariffs. Each model had a lifetime horizon (until the age of 100), an annual (hypertension, diabetes, anticoagulant), monthly (COPD), or weekly (asthma) cycle length with half-cycle correction and the UK Treasury-recommended 3.5% discount rate for both costs and outcomes, using cost-year 2014. Age-related mortality was included in each model. Entry age, disease severity, drug prescribed and health status in the models were those in the RCT cohort.

Table 3: Summary of probabilities in the Markov models in the adherent and non-adherent groups for each of the six models (for full details of parameter derivation for each model, see [22])

Parameter	Estimate and source
1 Hypertension-amlodipine	
P [stroke for adherent patient]	Age & sex dependent risk from ASCOT study [15]
P [MI/fatal CHD for adherent patient]	
P [stroke for non-adherent patient]	P [stroke for adherent patient] x effect of non-adherence (*HR*)
P [MI/fatal CHD for non-adherent patient]	P [MI/fatal CHD for adherent patient] x effect of non-adherence (*HR*)
Effect of non-adherence, *HR*	Intermediate vs. High adherence: HR= 1.39[23]
P [MI/CHD (non-fatal MI or fatal CHD) being fatal CHD]	Age & sex dependent risk from ASCOT study [15]
P [stroke being fatal]	
P [death for MI survivors}	
P [death for stroke survivors}	
P [death from all other causes]	Age and sex dependent [24]
2 Hypertension-ramipril	
P [fatal cardiovascular event (MI, stroke,coronary event, heart failure, other cardiovascular events, first events) for adherent patient]	0.0068 [16]
P [fatal cardiovascular event for non-adherent patient]	0.0083, P[fatal cardiovascular event for adherent patient] x effect of non-adherence (HR)]
P [non-fatal MI for adherent patient]	0.0041 [16]
P [non-fatal stroke for adherent patient]	0.0075 [16]
P [non-fatal MI for adherent patient]	0.0050: P[non-fatal MI for adherent patient] x effect of non-adherence (HR)]

Elliott RA, *et al. BMJ Qual Saf* 2020; 29:286–295. doi: 10.1136/bmjqs-2018-009177

Parameter	Estimate and source	
P [non-fatal stroke for adherent patient]	0.0092: P[non-fatal stroke for adherent patient] x effect of non-adherence (HR)]	
P [death for MI survivors}	Age, sex, number of years from MI dependent, [25] personal communication	
P [death for stroke survivors}	Age, sex, number of years from stroke dependent, [26] personal communication	
Effect of non-adherence, *HR*	HRadh = 0.81 (95% CI: 0.67 – 0.98), adjusted for age and sex (base case scenario) [27]	
P [death from all other causes]	Age and sex dependent [24]	
3 Asthma-beclometasone model		
	Adherent patients[28],[29]	Non-adherent patients [28],[29]
P [Successful control⇨ Sub-optimal control]	0.1563 x (1-p) [a]	0.3710 x (1-p)[30 a]
P [Successful control⇨ Primary care exacerbation]	0.0135 x (1-p) [a]	
P [Successful control⇨ Secondary care exacerbation]	0.0054 x (1-p) [a]	
P [Sub-optimal control⇨ Successful control]	0.1394 x (1-p) [a]	
P [Sub-optimal control⇨ Sub-optimal control]	0.8322 x (1-p) [a]	
P [Sub-optimal control⇨ Primary care exacerbation]	0.0174 x (1-p) [a]	
P [Sub-optimal control⇨ Secondary care exacerbation]	0.0109 x (1-p) [a]	
P [Secondary care exacerbation ⇨ Successful control]	0.2000 x (1-p) [a]	
P [Secondary care exacerbation ⇨ Sub-optimal control]	0.2000 x (1-p) [a]	
P [Secondary care exacerbation ⇨ Primary care exacerbation]	0.4000 x (1-p) [a]	
P [Secondary care exacerbation ⇨ Secondary care exacerbation]	0.4000 x (1-p) [a]	
P [death from all other causes]	Age and sex dependent [24]	
4 Chronic obstructive pulmonary disease-Tiotropium model		
Probability of exacerbation	Adherent patients[31 32]	Non-adherent patients [31 32]
Moderate COPD ⇨ exacerbation	0.051 (SE: 0.004)	0.089
Moderate COPD ⇨ severe exacerbation given an exacerbation occurs	0.097 (SE: 0.024)	0.165
Severe COPD ⇨ exacerbation	0.075 (SE: 0.003)	0.129
Severe COPD ⇨ severe exacerbation given an exacerbation occurs	0.136 (SE: 0.018)	0.229
Very severe COPD ⇨ exacerbation	0.096 (SE: 0.005)	0.164
Very severe COPD ⇨ severe exacerbation given an exacerbation occurs	0.192 (SE: 0.027)	0.316
Effect of non-adherence on exacerbations rate, HR	44% (HR:0.56;95%CI:0.48-0.65) lower rate of severe exacerbations for adherent patients [32]	
Effect of non-adherence on death rate, HR	60% (HR: 0.4; 95%CI:0.35-0.46) lower risk of death for adherent patients[32]	
P [moving between chronic health states] Year 1, subsequent years	[31]	

Parameter	Estimate and source	
5 Diabetes metformin model		
P[fatal first diabetes complication (MI, CHF, stroke, renal failure, amputation) for adherent patient]	Patient characteristic and HbA1c dependent value from UKPDS68 [19] and UKPDS34[33]	
P[fatal first diabetes complication for nonadherent patient]		
P[non-fatal first diabetes complication (MI, CHF, stroke, renal failure, amputation, blindness, IHD) for adherent patient]		
P[non-fatal first diabetes complication for nonadherent patient]		
P[second non-fatal diabetes complication after the first complication]		
P [death from all other causes]	Age and sex dependent [24]	
Effect of non-adherence	HbA1c multiplier for non-adherent patient: 1.105 (95%CI: 1.047, 1.166)<br>HbA1c for non-adherent patient (age) = HbA1c for adherent patient (age) x 1.105[34]	
6 Antiplatelets/anticoagulants aspirin model		
	Adherent patient	Non-adherent patient
P [Event-free → Non-fatal MI[b]]	0.0056; 0.0070, 0.0089	0.0086; 0.0107, 0.0136[20]
P [Event-free → Non-fatal stroke[b]]	0.0019; 0.0026; 0.0048	0.0028; 0.0038; 0.0070 [21]
P [Event-free → Fatal MI/CHD[b]]	0.0015; 0.0023; 0.0056	0.0023; 0.0035; 0.0086 [20]
P [Event-free → Fatal stroke[b]]	0.0002; 0.0003; 0.0021	0.0003; 0.0004; 0.0031 [21]
P [Non-fatal MI → Death]	Dependent on age, year after the first MI, sex[25]	
P [Non-fatal stroke→ Death]	Dependent on age, year after the first stroke, sex [26]	
P [death from all other causes]	Age and sex dependent [24]	

[a]Transition probabilities between asthma states from [28],[29] with mortality (P[death from all other causes]) incorporated (p)

[b]Probabilities for 3 age-groups, 50-64 years, 65-74, and 75-84 years, respectively. Probabilities calculated from incidence rates reported. In the case of non-adherent patients, incident rates adjusted by the effect of non-adherence (reported rate ratios for events, comparing non-adherence vs. adherence).

BMI: body mass index; CHD: coronary heart disease; CHF: congestive heart failure; CI: confidence intervals; COPD: chronic obstructive pulmonary disease; HbA1c: glycosylated haemoglobin; HDL: high density lipoprotein; HR: hazard ratio; IHD: ischaemic heart disease; MI: myocardial infarction; p: probability; SBP: systolic blood pressure; se: standard error; UKPDS: United Kingdom Prospective Diabetes Study.

Elliott RA, *et al. BMJ Qual Saf* 2020; 29:286–295. doi: 10.1136/bmjqs-2018-009177

Table 4 Summary of utilities and costs for the Markov models in the adherent and non-adherent groups for each of the six models (for full details of parameter derivation for each model, see[22])

Health state	Utility weights	Mean cost/patient [£; 2014 values]
1 Hypertension-amlodipine		
**WELL**	Age & sex dependent no cardiovascular event [35]	Mean annual cost of medication (amlodipine): 13.4 [36 37]
**Non-fatal MI**	Age & sex dependent + MI history Utility decrement (MI) added [35]	1st year: 5704.6<br>≥2nd year: 986.7 [38 39 36 37]
**Non-fatal stroke**	Age & sex dependent + stroke history Utility decrement (stroke) added [35]	1st year: 4161.8<br>≥2nd year: 770.9 [38 39 36 37]
2 Hypertension-ramipril		
**WELL**	Age & sex dependent no cardiovascular event [35]	Mean annual cost of medication (ramipril): 95.8[36 37]
**Non-fatal MI***	Age and & dependent + MI history Utility decrement (MI) [35]	1st year: 5787<br>≥2nd year: 1069
**Non-fatal stroke***	Age & sex dependent + stroke history, Utility decrement (stroke) added [35]	1st year: 4244<br>≥2nd year: 853
3 Asthma-beclometasone		
**Successful control**	0.900 [40 41]	13.4 [17 39]
**Sub-optimal control**	0.842 [40 41]	34.9 [17 39]
**Asthma exacerbation**	Primary care-managed: 0.57<br>Hospital-managed: 0.33 [42]	Primary care-managed: 105.6<br>Hospital-managed: 2013.1 [28 39]
4 COPD-tiotropium		
**Moderate COPD**	0.787[101, 103]	46.53 per month [43]
**Severe COPD**	0.750 [101, 103]	79.32 per month[43]
**Very severe COPD**	0.647 [101, 103]	125.13 per month[43]
**COPD exacerbation**	Non-severe decrement: 0.01<br>Severe decrement: 0.042 [44 45]	Non-severe: 75.97<br>Severe: 1372[43]
5 Diabetes-metformin		
**Well**	Age and sex dependent no cardiovascular event [35]	Mean annual cost of medication (metformin): 8.05[36 37]
**Other diabetes health states**	*Utility decrement [46]*	*Fatal event; Non-fatal event 1st year; Non-fatal event ≥2nd year [39 46 47]*
**IHD**	-0.090	N/A; 2916.4; 963.8
**MI**	-0.055	1477.7; 5624.0; 926.0
**CHF**	-0.108	3252.8; 3252.8; 1140.2
**Stroke**	-0.164	4338.9;3440.0; 650.1
**Amputation**	-0.280	11200.4; 11200.4; 646.9
**Blindness**	-0.074	N/A; 1469.0; 622.0
**Renal failure**	-0.263	32452.5; 32452.5; 32452.5
6 Antiplatelets/anticoagulants-aspirin		
**Event-free**	Age & sex dependent+utility decrement for MI/stroke history [35 20]	1510.9[38 39 36 37 35 20]
**Non-fatal MI**	Age & sex dependent + utility decrement for MI [35 20]	1st year after MI: 6662.5<br>≥2nd year: 1597.1 [38 35-37 39 20]

Elliott RA, et al. BMJ Qual Saf 2020; 29:286–295. doi: 10.1136/bmjqs-2018-009177

Health state	Utility weights	Mean cost/patient [£; 2014 values]
**Non-fatal stroke**	Age & sex dependent + utility decrement for stroke [35 21]	1st year after stroke: 4593.5<br>≥2nd year:1817.5 [38 36 37 39 35 21]

COPD: chronic obstructive pulmonary disease; IHD: ischaemic heart disease; MI: myocardial infarction; N/A: not applicable; p: probability

Elliott RA, *et al. BMJ Qual Saf* 2020; 29:286–295. doi: 10.1136/bmjqs-2018-009177

Table 5 NHS and non-NHS costs for normal practice and NMS intervention at 26 week follow-up

Cost category	Current practice (n=127)<br>Mean cost/£ (N, se)	NMS (n=135)<br>Mean cost/£ (N, se)
**Primary care total**	**121.16(122, 7.93)**	**117. 12(128, 8.49)**
GP total	99.23(106, 7.39)	96.90(119,7.50)
GP contact	89.15(102,6.71)	90.41(113,7.28)
GP home visit	2.68(2,2.11)	1.89(3,1.08)
GP phone call	7.40(26,1.53)	4.59(18,1.43)
Nursing total	21.94(100, 2.01)	20.23(96,2.23)
nurse contact	19.71(97,1.90)	18.28(95,2.03)
nurse home visit	1.69(5,0.86)	1.59(4,0.93)
nurse phone call	0.54(9,0.30)	0.36(10,0.12)
**Secondary care total**	390.75(76,58.38)	293.36(74,44.12)
Outpatient	212.91(71,31.37)	181.70(68,25.11)
Accident & Emergency	8.30(5,4.18)	8.68(3,5.73)
Daycase	133.97(23,30.47)	102.99(23,27.73)
Inpatient	35.56(2,27.01)	0(0,0)
**Allied HCP (NHS) total[a]**	8.30(27,2.34)	5.36(23,1.77)
Allied HCP contact	5.42(26,1.34)	4.16(19,1.57)
Allied HCP home visit	2.76(4,1.56)	1.09(3,0.69)
Allied HCP phone call	0.11(2,0.07)	0.11(3,0.08)
**NMS intervention**	**0**	**24.60**
**Total NHS cost**	**520.21(126, 62.04)***	**415.84(132, 46.45)**
**Community based practitioner total[b]**	7.09(8,3.49)	11.67(2,11.42)
Community based practitioner phone call	0.15(4,0.07)	0.11(1,0.11)
Community based practitioner contact	1.18(3,0.84)	0.25(1,0.25)
Community based practitioner home visit	5.75(5,3.06)	11.32(1,11.32)
**Allied HCPs non-NHS total**	11.25(54,1.71)	11.51(74,1.44)
Community pharmacist	9.66(49,1.55)	9.31(66,1.26)
Other associated HCPs non-NHS[c]	1.58(13,0.47)	2.20(20,0.57)
**Total non-NHS cost**	**18.33(58,3.92)**	**23.18(74,11.71)**

[a]Allied health care professionals (NHS) include: podiatrists, phlebotomists; [b]Community based practitioners include: social workers; [c]Allied health care professionals (non-NHS) include: dentists, opticians, chiropractors.
*Mean difference in costs: £104.36 (95% CI:-37.84- 256.52, p=0.168, z value:1.38)
GP: general practitioner; HCP: health care practitioner; NHS: National Health Service, se: standard error

Economic analysis

Elliott RA, *et al. BMJ Qual Saf* 2020; 29:286–295. doi: 10.1136/bmjqs-2018-009177

The deterministic analysis reported that NMS generated a mean of 0.04 more QALYs per patient, at a mean reduced cost of -£108.9. The probabilistic analysis reported very similar results, that NMS generated a mean of 0.04 (95% CI: -0.01, 0.13) more QALYs per patient, at a mean reduced cost of -£113.9 (95% CI: -1159.4, 683.7), see Table 7.

Table 7 Incremental economic analysis of NMS versus normal practice at 26-week follow-up: deterministic and probabilistic analysis

Incremental analysis	Mean cost (£)		Mean QALY		Incremental		ICER (£/QALY)
	NMS*	Normal practice	NMS	Normal practice	Cost/£	QALY	
Deterministic	19 102.2	19 211.2	13.47	13.43	-108.9	0.04	-2 758.4
Probabilistic (95% CI**)	20 482.7 (9 438.9, 53 822.0)	20 596.5 (9 435.5, 54 125.5)	13.45 (12.55, 14.35)	13.41 (12.50, 14.31)	-113.9 (- 1 159.4, 683.7)	0.04 (-0.01, 0.13)	-2 847.5

*Incorporating cost of intervention equal to £24.6
**95% CIs were obtained from 2.5% and 97.5% percentiles for costs, QALYs and ICERs in probabilistic sensitivity analysis.
ICER: incremental cost effectiveness ratio; QALY: quality-adjusted life-year

Elliott RA, *et al. BMJ Qual Saf* 2020; 29:286–295. doi: 10.1136/bmjqs-2018-009177

Appendix 6 Marginal probabilities for Models 1 and 2 for primary and secondary outcomes at 26 week follow-up

ITT at week 26 follow-up	Model 1 probability (95% CI)	Model* 2 probability (95% CI)
Adherence NMS (n=327)		
Current practice	0.57 (0.50, 0.65)	0.60 (0.52, 0.68)
NMS	0.66 (0.58, 0.73)	0.69 (0.62, 0.77)
Adherence MMAS 8 (n=223)		
Current practice	0.56 (0.48, 0.65)	0.63 (0.52, 0.74)
NMS	0.64 (0.54, 0.73)	0.73 (0.63, 0.83)

Elliott RA, *et al. BMJ Qual Saf* 2020; 29:286–295. doi: 10.1136/bmjqs-2018-009177

References

1. Price VE, Blanchette VS, Ford-Jones EL. The Prevention and Management of Infections in Children with Asplenia or Hyposplenia. *Infectious Disease Clinics of North America21(3)()(pp 697-710), 2007Date of Publication: Sep 2007* 2007(3):697-710.
2. Office of Fair Trading. Evaluating the impact of the 2003 OFT study on the Control of Entry regulations in the retail pharmacies market. In: Trading OoF, ed. London, 2010.
3. Department for Environment Food and Rural Affairs. Archive: Rural definition and local authority classification. 2010 [Available from: http://archive.defra.gov.uk/evidence/statistics/rural/rural-definition.htm (Accessed 16/08/2012).
4. Office for National Statistics. Rural and Urban Area Classification for Super Output Areas, 2004, 2004:Rural/Urban Classifications.
5. Social Disadvantage Research Centre at the University of Oxford. The English Indices of Deprivation 2010. In: Government DfCaL, ed., 2010.
6. Office for National Statistics. Enumeration Postcodes (2011) to output areas (2011) to lower layer super output areas (2011) to middle layer super output areas (2011) to local authority districts (2011) E+W Lookup, 2011.
7. Elliott RA, Boyd M, Waring J, et al. Understanding and Appraising the New Medicines Service in the NHS in England (029/0124)' A randomised controlled trial and economic evaluation with qualitative appraisal comparing the effectiveness and cost effectiveness of the New Medicine Service in community pharmacies in England University of Nottingham 2014 [Available from: http://www.nottingham.ac.uk/~pazmjb/nms/downloads/report/files/assets/common/downloads/108842%20A4%20Main%20Report.v4.pdf accessed 10/10/15.
8. SAS Institute Inc. SAS Version 9.3(TS1M1). SAS Institute Inc., Cray, NC, USA, 2011.
9. Elliott RA. Poor adherence to anti-inflammatory medication in asthma: reasons, challenges, and strategies for improved disease management. *Dis-Manage-Health-Outcomes* 2006;14(4):223-33.
10. Personal Social Services Research Unit (PSSRU). Unit Costs of Health and Social Care. In: Curtis L, ed., 2012.
11. Personal Social Services Research Unit (PSSRU). Unit costs of health and social care. . In: Curtis L, ed., 2010.
12. McCann L, Hughes CM, Adair CG. A self-reported work-sampling study in community pharmacy practice: A 2009 update. *Pharmacy World and Science* 2010;32:536-43. doi: http://dx.doi.org/10.1007/s11096-010-9405-x
13. Moran A, Nancarrow S, Enderby P, et al. Are we using support workers effectively? The relationship between patient and team characteristics and support worker utilisation in older people's community-based rehabilitation services in England. *Health & Social Care in the Community* 2012;20(5):537-49. doi: 10.1111/j.1365-2524.2012.01065.x
14. Health and Social Care Information Centre. Hospital Episode Statistics 2013 [Available from: http://www.hscic.gov.uk/hes accessed 29/04/2103.
15. Lindgren P, Buxton M, Kahan T, et al. The Lifetime Cost Effectiveness of Amlodipine-Based Therapy Plus Atorvastatin Compared with Atenolol Plus Atorvastatin, Amlodipine-Based Therapy Alone and Atenolol-Based Therapy Alone: Results from ASCOT1. *Pharmacoeconomics* 2009;27(3):221-30.
16. Wing LMH, Reid CM, Ryan P, et al. A Comparison of Outcomes with Angiotensin-Converting–Enzyme Inhibitors and Diuretics for Hypertension in the Elderly. *New England Journal of Medicine* 2003;348(7):583-92. doi: doi:10.1056/NEJMoa021716

Elliott RA, *et al. BMJ Qual Saf* 2020; 29:286–295. doi: 10.1136/bmjqs-2018-009177

17. Steuten L, Palmer S, Vrijhoef B, et al. Cost-utility of a disease management program for patients with asthma. *International Journal of Technology Assessment in Health Care* 2007;23(2):184-91.
18. Price D, Asukai Y, Ananthapavan J, et al. A UK-Based Cost-Utility Analysis of Indacaterol, A Once-Daily Maintenance Bronchodilator for Patients with COPD, Using Real World Evidence on Resource Use. *Appl Health Econ Health Policy* 2013;11(3):259-74. doi: 10.1007/s40258-013-0021-5
19. Clarke PM, Gray AM, Briggs A, et al. A model to estimate the lifetime health outcomes of patients with Type 2 diabetes: the United Kingdom Prospective Diabetes Study (UKPDS) Outcomes Model (UKPDS no. 68). *Diabetologia* 2004;47(10):1747-59. doi: 10.1007/s00125-004-1527-z
20. Rodríguez LAG, Cea-Soriano L, Martín-Merino E, et al. Discontinuation of low dose aspirin and risk of myocardial infarction: case-control study in UK primary care. *BMJ* 2011;343 doi: 10.1136/bmj.d4094
21. Garcia Rodriguez LA, Cea Soriano L, Hill C, et al. Increased risk of stroke after discontinuation of acetylsalicylic acid: A UK primary care study. *Neurology* 2011;76(8):740-46.
22. Elliott RA, Tanajewski L, Gkountouras G, et al. Cost Effectiveness of Support for People Starting a New Medication for a Long-Term Condition Through Community Pharmacies: An Economic Evaluation of the New Medicine Service (NMS) Compared with Normal Practice. *Pharmacoeconomics* 2017;35:1237-55. doi: 10.1007/s40273-017-0554-9 [published Online First: 2017/08/05]
23. Mazzaglia G., Ambrosioni E., Alacqua M., et al. Adherence to Antihypertensive Medications and Cardiovascular Morbidity Among Newly Diagnosed Hypertensive Patients. *Circulation* 2009;120:1598-605.
24. Office for National Statistics. Mortality rates UK 2009-2010 2012 [Available from: http://www.ons.gov.uk/ons/taxonomy/search/index.html?nscl=Life+Tables&nscl-orig=Life+Tables&content-type=Dataset&content-type=Reference+table&sortDirection=DESCENDING&sortBy=pubdate.
25. Smolina K, Wright FL, Rayner M, et al. Long-Term Survival and Recurrence After Acute Myocardial Infarction in England, 2004 to 2010. *Circulation: Cardiovascular Quality and Outcomes* 2012 doi: 10.1161/circoutcomes.111.964700
26. Luengo-Fernandez R, Gray AM, Rothwell PM, et al. A population-based study of hospital care costs during 5 years after transient ischemic attack and stroke. *Stroke; a journal of cerebral circulation* 2012;43(12):3343-51.
27. Nelson R, Reid CM, Ryan P, et al. Self-reported adherence with medication and cardiovascular disease outcomes in the Second Australian National Blood Pressure Study (ANBP2). *Medical Journal of Australia* 2006;185:487-89.
28. Price MJ, Briggs AH. Development of an economic model to assess the cost effectiveness of asthma management strategies. *Pharmacoeconomics* 2002;20(3):183-94.
29. Kavuru M, Melamed J, Gross G, et al. Salmeterol and fluticasone propionate combined in a new powder inhalation device for the treatment of asthma: a randomized, double-blind, placebo-controlled trial. *The Journal of allergy and clinical immunology* 2000;105(6 Pt 1):1108-16. [published Online First: 2000/06/16]
30. Murphy AL. The community pharmacy SIMPLE approach to asthma management. Regional Innovation Fund (RIF) Project Evaluation Report., 2012.
31. Rutten-van Mölken MMH, Oostenbrink J, Miravitlles M, et al. Modelling the 5-year cost effectiveness of tiotropium, salmeterol and ipratropium for the treatment of chronic obstructive pulmonary disease in Spain. *The European Journal of Health Economics* 2007;8(2):123-35. doi: 10.1007/s10198-007-0039-4

32. Vestbo J, Anderson JA, Calverley PMA, et al. Adherence to inhaled therapy, mortality and hospital admission in COPD. *Thorax* 2009;64(11):939-43. doi: 10.1136/thx.2009.113662
33. UK Prospective Diabetes Study (UKPDS) Group. Effect of intensive blood-glucose control with metformin on complications in overweight patients with type 2 diabetes (UKPDS 34). *Lancet* 1998;352(9131):854-65. [published Online First: 1998/09/22]
34. Krapek K, King K, Warren SS, et al. Medication adherence and associated hemoglobin A1c in type 2 diabetes. *Ann Pharmacother* 2004;38(9):1357-62. doi: 10.1345/aph.1D612 [published Online First: 2004/07/09]
35. Ara R, Brazier J. Health related quality of life by age, gender and history of cardiovascular disease: results from the Health Survey for England. Discussion Paper. In: WhiteRose Research Online, ed.: Universities of York, Sheffield, Leeds., 2009.
36. Pharmaceutical Services Negotiating Committee. Evaluation of Evidence Provided by PharmOutcomes New Medicines Service Data. , 2013.
37. British Medical Association, Royal Pharmaceutical Society of Great Britain. British National Formulary 66th Edition. September (66) ed. London: BMJ Group and RPS Publishing 2013.
38. Lindgren P, Buxton M, Kahan T, et al. Economic evaluation of ASCOT-BPLA: antihypertensive treatment with an amlodipine-based regimen is cost effective compared with an atenolol-based regimen. *Heart* 2008;94(2):e4. doi: 10.1136/hrt.2007.127217
39. Department of Health. HSHC 2013. Health Service Cost Index, Annual Summaries 2013 [Available from: http://www.info.doh.gov.uk/doh/finman.nsf/Newsletters?OpenView&Start=13.1&ExpandView.
40. Briggs AH, Bousquet J, Wallace MV, et al. Cost-effectiveness of asthma control: an economic appraisal of the GOAL study. *Allergy* 2006;61(5):531-36. doi: 10.1111/j.1398-9995.2006.01038.x
41. Bateman ED, Bousquet J, Keech ML, et al. The correlation between asthma control and health status: the GOAL study. *European Respiratory Journal* 2007;29(1):56-62. doi: 10.1183/09031936.00128505
42. Lloyd A, Price D, Brown R. The impact of asthma exacerbations on health-related quality of life in moderate to severe asthma patients in the UK. *Prim Care Respir J* 2007;16(1):22-7.
43. Hertel N, Kotchie RW, Samyshkin Y, et al. Cost-effectiveness of available treatment options for patients suffering from severe COPD in the UK: a fully incremental analysis. *International journal of chronic obstructive pulmonary disease* 2012;7:183-99. doi: 10.2147/copd.s29820 [published Online First: 2012/04/14]
44. Rutten-van Mölken MMH, Hoogendoorn M, Lamers L. Holistic Preferences for 1-Year Health Profiles Describing Fluctuations in Health. *Pharmacoeconomics* 2009;27(6):465-77. doi: 10.2165/00019053-200927060-00003
45. Hettle R, Wouters H, Ayres J, et al. Cost-utility analysis of tiotropium versus usual care in patients with COPD in the UK and Belgium. *Respiratory Medicine* 2012;106(12):1722-33. doi: http://dx.doi.org/10.1016/j.rmed.2012.09.006
46. Diabetes Trial Unit. UKPDS Outcomes Model: University of Oxford; 2013 [Available from: http://www.dtu.ox.ac.uk/outcomesmodel/.
47. Clarke P, Gray A, Legood R, et al. The impact of diabetes-related complications on healthcare costs: Results from the United Kingdom Prospective Diabetes Study (UKPDS Study No. 65). *Diabetic Medicine* 2003;20(6):442-50. doi: http://dx.doi.org/10.1046/j.1464-5491.2003.00972.x
